# Development of a Dynamic Diagnosis Grading System for Infertility Using Machine Learning

**DOI:** 10.1001/jamanetworkopen.2020.23654

**Published:** 2020-11-09

**Authors:** ShuJie Liao, Wei Pan, Wan-qiang Dai, Lei Jin, Ge Huang, Renjie Wang, Cheng Hu, Wulin Pan, Haiting Tu

**Affiliations:** 1 Department of Obstetrics and Gynecology, Tongji Hospital, Tongji Medical College, Huazhong University of Science and Technology, Wuhan, Hubei, China; 2 School of Applied Economics, Renmin University of China, Beijing, China; 3 School of Economics and Management, Wuhan University, Wuhan, China

## Abstract

**Importance:**

Many indicators need to be considered when judging the condition of patients with infertility, which makes diagnosis and treatment complicated.

**Objective:**

To construct a dynamic scoring system for infertility to assist clinicians in efficiently and accurately assessing the condition of patients with infertility.

**Design, Setting, and Participants:**

This prognostic study reviewed 95 868 medical records of couples with infertility in which women had undergone in vitro fertilization and embryo transfer at the Reproductive Center of Tongji Medical College, Huazhong University of Science and Technology, in Wuhan, Hubei, China, from January 2006 to May 2019. A dynamic diagnosis and grading system for infertility was constructed. The analysis was conducted between May 20, 2019, and April 15, 2020.

**Main Outcomes and Measures:**

Patients were divided into pregnant and nonpregnant groups according to eventual pregnancy results. The evaluation index system was constructed based on the test results of the significant difference between the 2 groups of indicators and the clinician’s experience. Random forest machine learning was used to determine the weight of the index, and the entropy-based feature discretization algorithm classified the abnormality of the index and the patient's condition. A 10-fold cross-validation method was used to test the validity of the system.

**Results:**

A total of 60 648 couples with infertility were enrolled, in which 15 021 women became pregnant, with a mean (SD) age of 30.30 (4.02) years. A total of 45 627 couples were in the nonpregnant group, with a mean (SD) age among women of 32.17 (5.58) years. Seven indicators were selected to build the dynamic grading system for patients with infertility: age, body mass index, follicle-stimulating hormone level, antral follicle count, anti-Mullerian hormone level, number of oocytes, and endometrial thickness. The importance weight of each indicator obtained by the random forest algorithm was 0.1748 for age, 0.0785 for body mass index, 0.0581 for follicle-stimulating hormone level, 0.1214 for antral follicle count, 0.1616 for anti-Mullerian hormone level, 0.2307 for number of oocytes, and 0.1749 for endometrial thickness. The grading system divided the condition of the patient with infertility into 5 grades from A to E. The worst E grade represented a 0.90% pregnancy rate, and the pregnancy rate in the A grade was 53.82%. The cross-validation results showed that the stability of the system was 95.94% (95% CI, 95.14%-96.74%).

**Conclusions and Relevance:**

This machine learning–derived algorithm may assist clinicians in making an efficient and accurate initial judgment on the condition of patients with infertility.

## Key Points

**Question:**

Can machine learning be used to establish a dynamic scoring system to assist clinicians in predicting the severity of infertility in patients?

**Findings:**

In this prognostic study using a dynamic scoring system established based on the medical records of 60 648 couples with infertility in which women underwent in vitro fertilization and embryo transfer, the overall stability test result of the system was 95.94%.

**Meaning:**

This machine learning–derived algorithm may assist clinicians in making an efficient and accurate initial judgment on the condition of patients with infertility.

## Teaser

This prognostic study assesses whether machine learning can be used to develop a dynamic scoring system for predicting the severity of infertility in patients.

## Introduction

Infertility has attracted attention worldwide. Infertility is defined as failure to achieve pregnancy within 12 months of unprotected intercourse or therapeutic donor insemination in women younger than 35 years or within 6 months in women older than 35 years.^1^ It is estimated that 1 in 6 couples in the world experiences infertility.^2^ Patients with infertility often experience psychological stress and are at risk for depression, cancer, and other diseases.^3,4^ However, the development of assisted reproductive technology (ART) has brought hope to couples with infertility. According to the US Centers for Disease Control and Prevention 2017 Fertility Clinic Success Rates Report, there were 284 385 ART cycles performed at 448 reporting clinics in the US during 2017, resulting in 78 052 live-born infants.^5^ China has also made great efforts to treat infertility. At the end of 2018, there were 497 medical institutions in China that had been approved to provide ART. In recent years, the total number of cycles of ART has exceeded 1 million per year in China, and the number of infants born has exceeded 300 000.^6^ Moreover, the treatment of infertility needs to consider a number of factors, including age,^7^ body mass index (BMI),^8^ hormone levels, and ovarian reserve capacity.^9,10,11,12^ These various factors make diagnosis and treatment strategy selection complicated. In addition, it is difficult to have a unified standard for reference for these complex indicators because of the data differences in various studies on infertility.^10,11,12^ To solve these difficulties, this study used a dynamic scoring system based on artificial intelligence to measure and evaluate the various physical indicators of the condition of patients with infertility to help clinicians with prognosis for these patients.

In the medical field, scoring systems have been widely applied in the treatment of familial Mediterranean fever,^13^ cirrhosis,^14,15^ stroke,^16^ osteoarthritis,^17^ and other diseases. In the field of reproduction, a simple scoring system has been established based on demographic characteristics and initial ultrasonography variables to predict the likelihood of pregnancy.^18^ Some researchers have used the endometriosis fertility index to score patients and give corresponding fertility guidance.^19,20^ However, in view of many complex patient indicators and no unified indicator reference standard for infertility, few reliable grading systems can help clinicians make treatment decisions about ART.

When considering the number of indicators and unclear standards, the application of a traditional grading system has many limitations. However, feature-engineering^21^ technology can better mine features from the original data and provide a new way to solve for multiple indicators. An entropy-based algorithm can produce better discrimination and is widely used. A recent study^22^ proposed an entropy-based combination method to score loan credit. In clinical application, some researchers have proposed an automatic sleep scoring method by combining multiscale entropy features with information on sleep architecture.^23^ In addition, a variety of artificial intelligence methods, such as random forest and neural networks, can be used to further improve the availability and accuracy of scoring systems. One study^24^ built a scoring system for patients with cirrhosis based on a random forest algorithm. Another study^25^ built a prediction model of gastrointestinal bleeding with machine learning that was superior to the traditional clinical risk scoring system. In view of these studies, this analysis combined the entropy-based and random forest algorithm to construct a dynamic grading system for reproduction to describe the physical condition of patients with infertility and select more-effective treatments.

## Methods

### Data Source

For this prognostic study, we reviewed 95 868 medical records of couples with infertility in which women had undergone in vitro fertilization and embryo transfer at the Reproductive Center of Tongji Medical College, Huazhong University of Science and Technology, in Wuhan, Hubei, China, from January 2006 to May 2019. The indications for in vitro fertilization and embryo transfer were infertility due to tubal and cervical factors, unexplained infertility, endometriosis, and ovulatory dysfunction and sterility due to oligozoospermia and asthenospermia. The study was approved by the ethics committee of the Reproductive Medicine Center of Tongji Hospital, and the patients gave written informed consent before participating. The study followed the Standards for Reporting of Diagnostic Accuracy (STARD) reporting guideline.

Of the initial 95 868 medical records, 29 185 records of frozen embryo transfer data, 5843 records of resuscitation data, 120 records of egg donation data, and 72 records of double uterus data involving fresh embryo transfer were excluded. A total of 60 648 records of single uterus fresh embryo data were included in the study. All patients underwent a comprehensive diagnostic evaluation of infertility, including history and physical examination, hormone tests, and ultrasonography, including transvaginal ultrasonography.

### Study Design

**Figure 1.**
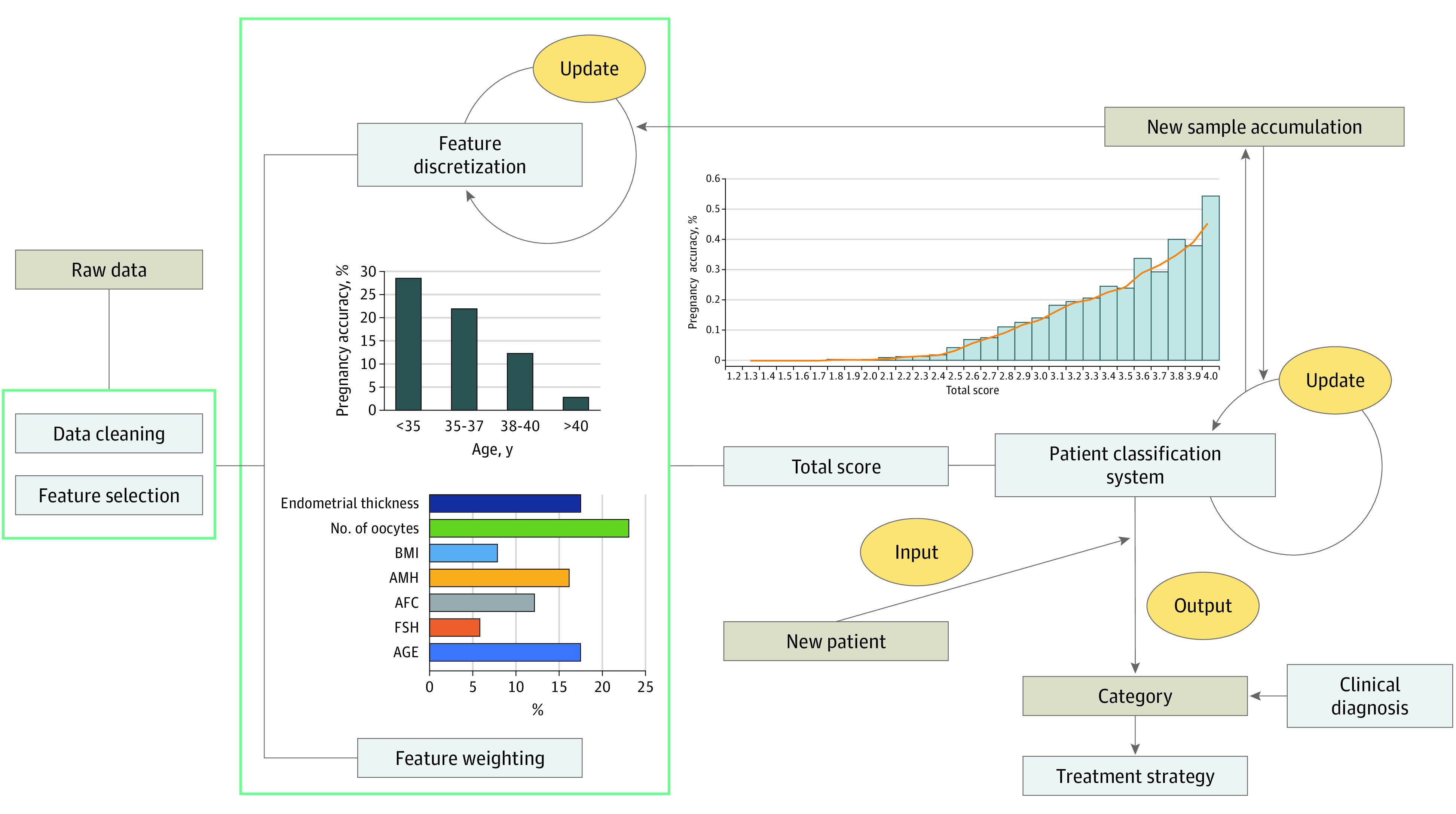
Flowchart of the Dynamic Grading System for Infertility The system construction process included 4 steps: (a) data cleaning and feature selection, (b) feature discretization and classification, (c) weight determination and total score calculation, and (d) system construction and testing. When new samples are added during this process, the system can complete automatic updates.

The flowchart of the dynamic grading system for infertility established in this study is shown in Figure 1. First, we removed or corrected obvious outliers caused by incorrect records in the sample according to the possible ranges of different indicators and filled in missing values according to the mode or mean. Second, through a 1-way analysis of variance between the pregnant group and the nonpregnant group, the index with *P* < .01 was selected and combined with clinicians and relevant literature to complete the index construction of the scoring system. Then, the entropy-based feature discretization algorithm was used to segment the selected key indicators and assign different categories to reflect abnormalities in the indicators of patients with infertility. Weights of each indicator were determined by the random forest algorithm. Finally, by segmenting the overall score for the patient, we constructed a complete dynamic grading system for infertility.

### Entropy-based Feature Discretization

Feature discretization is an important technique of feature engineering and is the basis of data interval division. Among discretization algorithms, entropy-based algorithms^26^ usually show better performance than other algorithms.^27,28^ In classification, class entropy is a measure of uncertainty in a finite interval of classes and can be used as an evaluation metric. The smaller the entropy, the smaller the uncertainty and the greater the data purity. An optimal partition should minimize the overall entropy of all subsets created. In practical terms, the class information entropy is calculated for all possible partitions and compared with the entropy without partitions. This can be done recursively until some stopping criterion is satisfied. The stopping criteria can be defined by a user or by a heuristic method such as Minimum Description Length Principle.^29^ The specific steps are given in eAppendix 1 in the Supplement.

### Random Forest Feature-Weighting Algorithm

Random forest^30^ is an ensemble learning algorithm composed of multiple decision trees. It uses mainly random resampling technology (bootstrap) to randomly extract a part of the data from the original sample to form a training set, and the remaining unextracted data are called *out-of-bag data*. Out-of-bag data are used mainly to test the generalization ability of the model and evaluate the importance of sample features. The specific steps are given in eAppendix 2 in the Supplement.

### Ten-fold Cross Validation

Ten-fold cross validation is a statistical analysis method that can be used to verify the performance of the classifier. In this method, the original data set is divided into 10 equal parts, 9 parts of which are used as training sets, with the remaining 1 part used as a test set. In this way, 10 models can be obtained, and the performance of the classifier is measured by the mean classification accuracy of these 10 models. Although the research in this study was not a dichotomy problem, the stability of the system could still be tested by using 10-fold cross validation. The specific steps are given in eAppendix 3 in the Supplement.

### Statistical Analysis

In the process of data analysis, R, version 3.6.2 (The R Project for Statistical Computing) was used to perform 1-way analysis of variance of the indicators, and Python, version 3.7.1 (Python) was used to complete the construction of the infertility dynamic grading system and cross-validation. In the index screening process, *P* < .01 was considered statistically significant, all tests were 2-tailed, and cross-validation used a 95% CI.

## Results

### Key Indicators Selected to Construct the System

A total of 60 648 medical records of couples with infertility who were included in the study were divided into 2 groups according to whether the patients had normal pregnancy characteristics in the sixth week after in vitro fertilization and embryo transfer (recheck if necessary); 15 021 were in the pregnant group (mean [SD] age of women, 30.30 [4.02] years), and 45 627 were in the non-pregnant group (mean [SD] age, 32.17 [5.58] years). The ratio of the 2 groups was 1 to 3.04. eTable 1 in the Supplement gives a detailed description of other patient characteristics.

Significant differences were found in many indicators between the 2 groups, including demographic characteristics, such as age, and hormone levels, such as follicle stimulating hormone level (FSH), anti-Mullerian hormone level (AMH), and ovarian reserve capacity indicators (antral follicle count [AFC], endometrial thickness). Specifically, compared with the nonpregnant group, the pregnant group had lower age (mean [SD]: 30.30 [4.02] years vs 32.17 [5.58] years; *P* < .01) and FSH level (mean [SD]: 6.99 [2.51] mIU/mL vs 7.75 [25.74] mIU/mL; *P* < .01), higher AFC (mean [SD]: 13.85 [5.32] vs 12.51 [6.39]; *P* < .01), and greater endometrial thickness (mean [SD]: 11.60 [2.31] mm vs 10.80 [3.05] mm; *P* < .01). There was no significant difference in mean (SD) BMI (calculated as weight in kilograms divided by height in meters squared) between pregnant (21.90 [2.31]) and nonpregnant (21.86 [1.94]) groups (*P* = .08). According to past research,^31^ we still included BMI as an indicator of the new dynamic grading system. Therefore, our indicator system included 7 indicators: age, BMI, FSH level, AFC, AMH level, number of oocytes, and endometrial thickness.

### Discretization Results of Indicators

**Figure 2.**
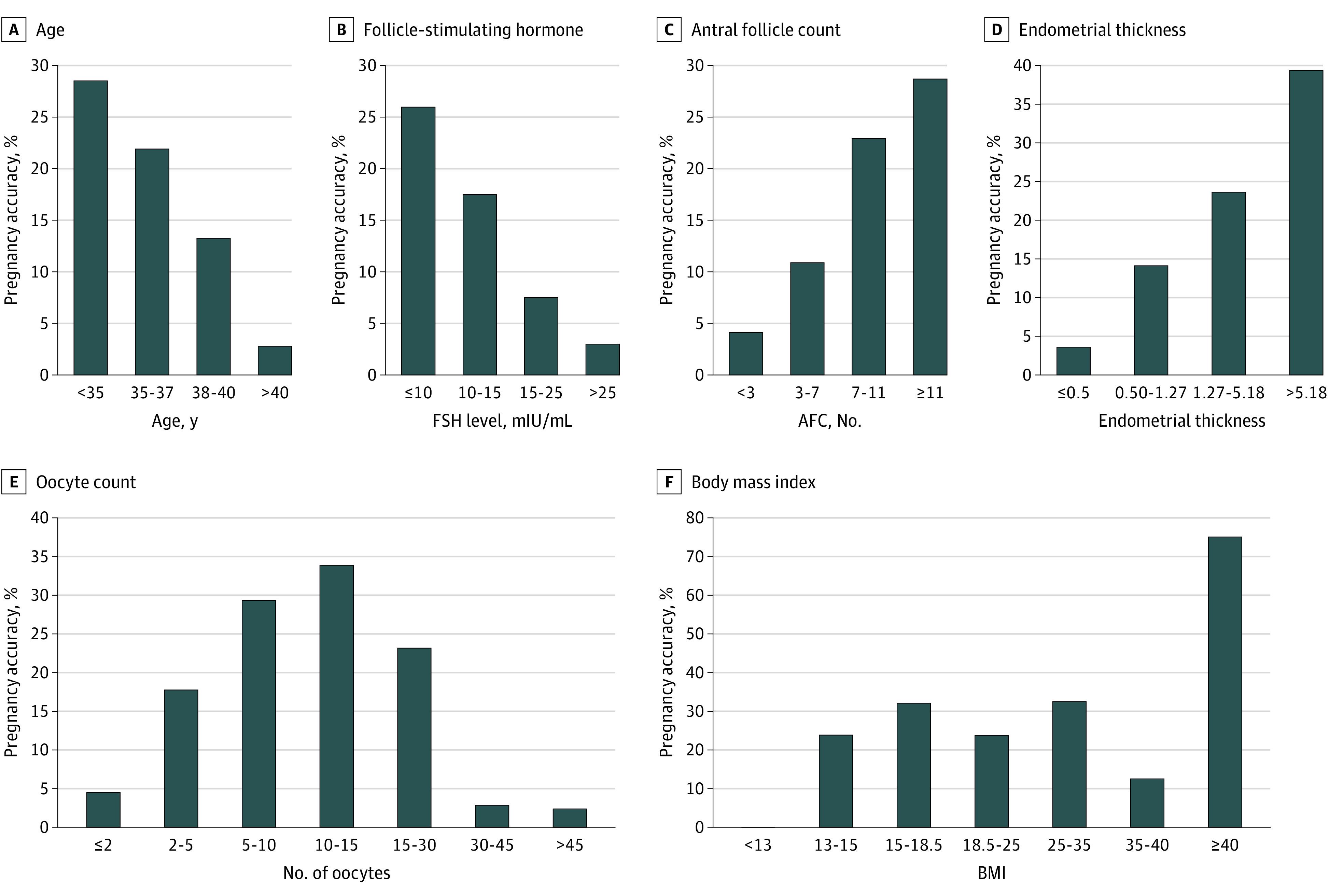
Interval Division Results of Key Indicators Body mass index (BMI) (calculated as weight in kilograms divided by height in meters squared) and number of oocytes are medium-sized indexes that are best when their eigenvalues are in the middle range. AFC indicates antral follicle count; AMH, anti-Mullerian hormone; FSH, follicle-stimulating hormone.

With use of the entropy-based feature discretization method, the aforementioned 7 indicators were divided into intervals (Figure 2 and eFigure 1 in the Supplement). Each feature was divided into 4 categories: A, B, C, and D, with 4 points, 3 points, 2 points, and 1 point assigned successively. The score of each category represents the degree of abnormality of the patient’s index. The lower the score, the more it deviates from the normal range. The pregnancy rate did not vary significantly by BMI, which made it difficult to perform segmentation through the entropy-based feature discretization algorithm. Therefore, we divided BMI according to the standards formulated by the World Health Organization. The normal range of BMI is 18.5 to 25, which was classified as grade A. BMI below or above this range was considered unhealthy, and the more the BMI deviated from this range, the lower the score.

### Weight of Each Indicator

With 80% of the samples as the training set and 20% as the test set, the random forest algorithm was used to assign corresponding weights to the 7 indicators. The weights of the indicators and the distribution of the number of patients in different categories are shown in the Table. The number of oocytes (weight, 23.07%), age (17.48%), endometrial thickness (17.49%), and AMH level (16.16%) had a stronger association with the pregnancy rate than did the other indicators. The number of oocytes and endometrial thickness reflect the capacity of ovarian reserve. Although FSH level (weight, 5.81%) and BMI (7.85%)had a weaker association with the pregnancy rate, they may still be important factors to consider in clinical practice.

**Table.** Grading and Weighting Results of 7 Indicators^a^

Indicators	Weight, %	Interval	Category	Score	Total sample	Pregnancy	
						Sample	Rate, %
Age, y	17.48	<35	A	4	44 523	12 698	28.52
		35-37	B	3	7246	1588	21.92
		38-40	C	2	4635	615	13.27
		>40	D	1	4243	119	2.80
FSH level, mIU/mL	5.81	≤10	A	4	53 973	14 015	25.97
		11-15	B	3	5157	903	17.51
		16-25	C	2	1250	94	7.52
		>25	D	1	267	8	3.00
AFC, No.	12.14	<3	D	1	1610	66	4.10
		3-6	C	2	8014	873	10.89
		7-10	B	3	9598	2199	22.91
		≥11	A	4	41 425	11 882	28.68
AMH level, ng/mL	16.16	≤0.50	A	4	1272	46	3.62
		0.51-1.27	B	3	2439	344	14.10
		1.28-5.18	C	2	49 398	11 660	23.60
		>5.18	D	1	7538	2970	39.40
BMI	7.85	<13.0	D	1	0	0	0.00
		13.0-14.9	C	2	21	5	23.81
		15.0-18.4	B	3	2962	951	32.11
		18.5-24.9	A	4	53 348	12 664	23.74
		25.0-34.9	B	3	4296	1395	32.47
		35.0-39.9	C	2	16	2	12.50
		≥40.0	D	1	4	3	75.00
Oocytes, No.	23.07	≤2	D	1	5176	233	4.50
		3-5	C	2	8269	1468	17.75
		6-10	B	3	16 355	4794	29.31
		11-15	A	4	14 796	5009	33.85
		16-30	B	3	15 063	3488	23.16
		31-45	C	2	946	27	2.85
		>45	D	1	42	1	2.38
Endometrial thickness, mm	17.49	≤6	A	1	1766	50	2.83
		7-8	B	2	5074	491	9.68
		9-11	C	3	18 947	4063	21.44
		≥11	D	4	34 860	10 416	29.88

Abbreviations: AFC, antral follicle count; AMH, anti-Mullerian hormone; BMI, body mass index (calculated as weight in kilograms divided by height in meters squared); FSH, follicle-stimulating hormone.

^a^ With use of the entropy-based feature discretization method, the 7 indicators were divided into intervals. Each feature was divided into 4 categories: A, B, C, and D, with 4 points, 3 points, 2 points, and 1 point assigned successively. The score of each category represents the degree of abnormality of the patient’s index. The lower the score, the more it deviates from the normal range (category A: normal [4 points]; category B: mildly abnormal [3 points]; category C: moderately abnormal [2 points]; category D: extremely abnormal [1 point]).

### A New Dynamic Diagnosis Grading System for Infertility

**Figure 3.**
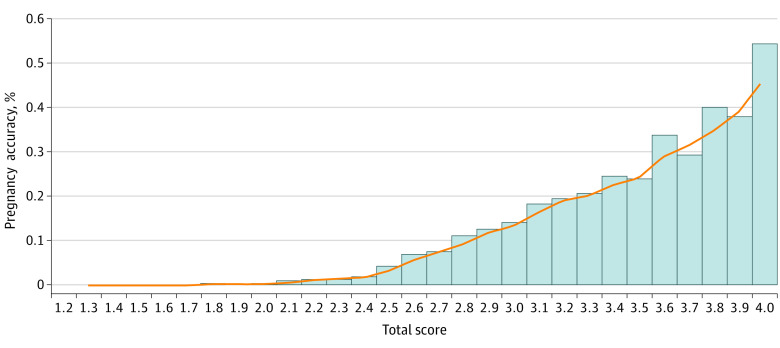
Pregnancy Rates and Total Scores for Patients The patient's comprehensive score was calculated by the weighted mean, and the interval distribution was 1 to 4 points.

**Figure 4.**
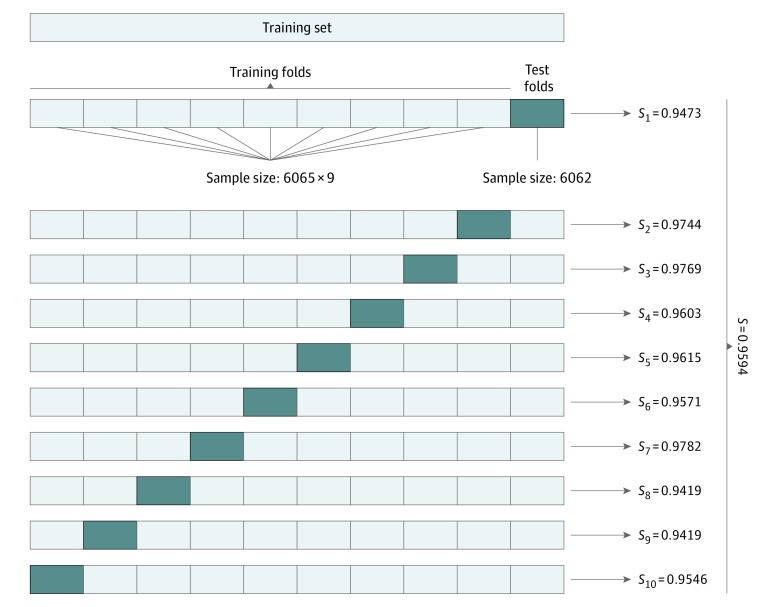
Ten-fold Cross Validation The total score data set of patients was divided into 10 approximately equal parts (6065 each except 6062 in the last group). *S* indicates sample.

By weighted summation of the score for each indicator, we developed a final comprehensive grading of the patients' condition. The association of the pregnancy rate with the final score is shown in Figure 3. A higher comprehensive score was associated with an increase in the pregnancy rate. The entropy-based method was also used to stratify the total score. Three stratification schemes were attempted (eTable 2 in the Supplement). According to the pregnancy rate among the patients in different divisions, we chose to divide the patients' conditions into 5 grades. When the final score for a patient with infertility was less than or equal to 2.38, she was classified into grade E, with a poor likelihood of pregnancy. A final score of greater than 3.84 indicated that the overall physical condition of the patient was good and the pregnancy rate was at least 53.82%. The results of 10-fold cross-validation are shown in Figure 4. The classification consistency of the system reached 95.94% (95% CI, 95.14%-96.74%).

### Association of Indicators With Pregnancy Rate

Based on the association of pregnancy rate with the change in indicator values (eFigure 2 in the Supplement), the key indicators included in the scoring system had the following characteristics. First, there was a slight upward trend in the pregnancy rate among patients aged 19 to 29 years, a slight downward trend among patients aged 29 to 34 years, and a substantial downward trend after 34 years of age. Second, higher FSH level was associated with a lower pregnancy rate. Third, when AFC ranged from 0 to 14, the pregnancy rate increased; when AFC was greater than 14, the pregnancy rate decreased slightly and then remained stable. Fourth, the pregnancy rate increased significantly when the AMH level was between 0 and 5 ng/mL and decreased when the AMH level was greater than 6 ng/mL. Fifth, there was no significant association between BMI and pregnancy rate. Sixth, the pregnancy rate increased when the number of oocytes was between 0 and 16 and decreased when the number of oocytes exceeded 16. The pregnancy rate was higher when the number of oocytes was 10 to 15. Seventh, when the endometrial thickness was less than 11 mm, there was a positive correlation between the endometrial thickness and pregnancy rate. Greater endometrial thickness was associated with a higher pregnancy rate. When the endometrial thickness was greater than 11 mm, the upward trend became stable.

## Discussion

To assist clinicians in having a comprehensive understanding of the physical condition of patients with infertility, this study used an entropy-based feature discretization algorithm and a random forest algorithm to build a new dynamic diagnosis grading system for infertility. To our knowledge, this is the first study to apply an artificial intelligence approach to the construction of a reproductive scoring system.

Following are key findings regarding the indicators. First, the pregnancy rate decreased with increasing age, which is consistent with previous research and clinical performance.^32^ Second, higher FSH was associated with a lower pregnancy rate. Previous studies have indicated that an FSH level less than or equal to 10 IU/L and an FSH level greater than 15 to 25 IU/L can be used to indicate standard normal and abnormal ovarian reserve function, respectively,^9^ which is consistent with findings of the present study. Third, lower AFC was associated with a slightly increased pregnancy rate, consistent with findings of previous studies.^29,33^

Fourth, the pregnancy rate increased significantly when the AMH level was between 0 and 5 ng/mL and decreased after the AMH level exceeded 6 ng/mL. The reason for the abnormal downward trend in the at an AMH level of 5 to 6 ng/mL may be that AMH level was affected by age and other factors or that the sample size of AMH (greater than 5 ng/mL) was less. However, in general, the positive correlation between AMH level and the pregnancy rate was consistent with findings of a prior study.^12^ Fifth, there was no significant association between BMI and pregnancy rate. Sixth, the pregnancy rate was higher when the number of oocytes was 10 to 15, which is consistent with findings of a previous study.^10^ Seventh, greater endometrial thickness was associated with a higher pregnancy rate. When the endometrial thickness exceeded 11 mm, the upward trend became stable, which is also consistent with findings of a previous study.^11^

This scoring system is not fixed and unchangeable. As the number of new samples increases, the model can be further verified to appropriately adjust and update the interval division boundary and the number of category divisions of each indicator feature. The real-time update process may lead to more efficient and accurate judgment about patients' conditions and assist clinicians in comprehensively and effectively understanding patients' conditions and formulating treatment plans.

### Limitations

This study has limitations. First, in the selection process of indicators, we did not consider the complex correlations among indicators (eg, the association of AMH level with age and other factors). Second, owing to the small sample size of individual indicators in certain ranges, there may be an abnormality between the interval of individual indicators and the pregnancy rate. The most obvious example is the high pregnancy rate when BMI was greater than 40 (only 4 samples, with 3 in the pregnant group). Third, the couples in which women underwent in vitro fertilization and embryo transfer were included from a large period between January 2006 and May 2019 at the Reproductive Center of Tongji Medical College, affiliated with Huazhong University of Science and Technology. Furthermore, this study used only the medical records of a single hospital as the research data. Even if the sample size was sufficiently large, there may be regional and population limitations.

## Conclusions

The new dynamic diagnosis grading system for infertility in this study may assist clinicians in making a quick and effective preliminary judgment of the condition of patients with infertility. Only relevant indicators of patients need to be input into the system to get the abnormal situation of each indicator and have a comprehensive understanding of the severity of the patient's condition so that the corresponding treatment of the abnormal indicators can be accounted for in making a more targeted treatment plan. In addition, this system was more accurate and practical than a previous single risk factor assessment^8,11,29^ because it assessed multiple physical indicators of patients comprehensively.

## Notes

Supplement. **eAppendix 1.** Entropy-based Feature Discretization Algorithm **eAppendix 2.** RF Feature Weighting Method **eAppendix 3.** 10-fold Cross Validation **eFigure 1.** Interval Division Result of Endometrial Thickness **eFigure 2.** The Relationship Between The Pregnancy Rate And the Seven Indicators **eTable 1.** Comparison Between Pregnant Group and the Non-pregnant Group **eTable 2.** Three Grading Schemes for Final Score of Patients
