# Error in Figure 2

**DOI:** 10.1001/jamanetworkopen.2025.37746

**Published:** 2025-09-19

In the Original Investigation titled “Development of a Dynamic Diagnosis Grading System for Infertility Using Machine Learning,” published on November 9, 2020, Figure 2D was updated to indicate that anti-Mullerian hormone levels, not endometrial thickness, are shown. This article has been corrected online.^1^
