# Supplementary material for: Development of a Dynamic Diagnosis Grading System for Infertility Using Machine Learning
Source: JAMA Netw Open. 2020 Nov 9;3(11):e2023654. doi: 10.1001/jamanetworkopen.2020.23654 (PMC7653500; doi:10.1001/jamanetworkopen.2020.23654)
Supplement: Supplement. — eAppendix 1. Entropy-based Feature Discretization Algorithm eAppendix 2. RF Feature Weighting Method eAppendix 3. 10-fold Cross Validation eFigure 1. Interval Division Result of Endometrial Thickness eFigure 2. The Relationship Between The Pregnancy Rate And the Seven Indicators eTable 1. Comparison Between Pregnant Group and the Non-pregnant Group eTable 2. Three Grading Schemes for Final Score of Patients [file jamanetwopen-e2023654-s001.pdf]

Supplemental Online Content

Liao S, Pan W, Dai WQ, et al. Development of a dynamic diagnosis grading system for infertility using machine learning. *JAMA Netw Open*. 2020;3(11):e2023654. doi:10.1001/jamanetworkopen.2020.23654

eAppendix 1. Entropy-based Feature Discretization Algorithm

eAppendix 2. RF Feature Weighting Method

eAppendix 3. 10-fold Cross Validation

eFigure 1. Interval Division Result of Endometrial Thickness

eFigure 2. The Relationship Between The Pregnancy Rate And the Seven Indicators

eTable 1. Comparison Between Pregnant Group and the Non-pregnant Group

eTable 2. Three Grading Schemes for Final Score of Patients

This supplemental material has been provided by the authors to give readers additional information about their work.

eAppendix 1. Entropy-based feature discretization algorithm

The specific steps of the Entropy-based feature discretization algorithm are as follows:

Step 1: Sort all sample values of feature A in ascending order;

Step 2: Traverse each sample value of feature A, and use each sample value as a segmentation point to divide the sample into 2 sample subsets;

Step 3: Calculate the weighted average entropy of the sample subset after each point is divided, and select the one with the smallest weighted average entropy as the segmentation point;

$$E(j) = -\sum_{i=1}^{n} p(x_i) log_2 P(x_i)$$

$$E = \sum_{j=1}^{k} \omega_j E(j)$$

$E(j)$: the entropy of the *j-th* $(j = 1, 2, \cdots, k)$ sample subset;

$P(x_i)$: the frequency of class $i\ (i = 1, 2, \cdots, n)$ labels in the sample subset;

ω_j: proportion of samples in *j-th* sample subset to total samples.

Step 4: When the entropy after division is greater than the set threshold and less than the specified number of groups, repeat steps 2-3 to continue the division, otherwise stop and output the division result.

There are two parameters that determine the effect of Entropy-based discretization segmentation. The first is the maximum number of groups of samples, that is, the number of intervals after discretization; the second is the minimum entropy that stops dividing. These two parameters together limit the segmentation to endless. For different characteristics, we used the method of repeated trials and combined with clinical experience to find the best classification parameters.

eAppendix 2. RF feature weighting method

The specific steps of the RF feature weighting method are as follows:

Step 1: Using bootstrap technology to generate multiple decision trees from the

© 2020 Liao S et al. *JAMA Network Open.*

original data set, construct a random forest model, determine the OOB data outside the bag, and calculate the data error *OBBerror1* according to the generated model.

Step 2: Randomly change the value of feature A in OOB data (i.e. the noise interference of feature A), and calculate the OOB data error *OBBerror2* again.

Step 3: Suppose there are n trees in the forest, and *feature importance = (error2-error1) / n*. The degree of feature importance is determined according to the magnitude of the numerical change. Finally, the importance of all features is normalized to obtain the weight of each feature.

For the features in the data, the variable with a larger significance score indicates that the variable is more important for classification and the corresponding weight is larger. The variable importance measurement is a natural mechanism behind RF simulation data, which has good statistical robustness and good application in different fields.

eAppendix 3. 10-fold cross validation

The specific steps of 10-fold cross validation are as follows:

Step 1: The total scores of all samples are randomly divided into 10 equal parts to obtain 10 sample subsets.

Step 2: 9 sample subsets are selected in turn to form the training set, and the remaining one is the test set. The total score of each training set is graded by Entropy-based method, and 10 grading systems are established after 10 divisions.

Step 3: Test the stability of each classification system using the test set corresponding to the training set, and define the stability index as follows:

$$S_n = 1 - \sum_{i=1}^{K} \left| p_i^n(x_1) - P_i^n(x_1) \right| \quad or \quad S_n = 1 - \sum_{i=1}^{K} \left| p_i^n(x_0) - P_i^n(x_0) \right|$$

S_n: The stability of the $n \ (n = 1, 2, \cdots, 10)$ classification system;

$p_i^n(x_1) / p_i^n(x_0)$: Frequency of label 1 (or 0) in grade $i(i = 1, 2, \cdots, K)$ of the n grading system;

Step 4: The average stability of all grading systems after 10 cross validation is calculated to measure the final stability of the whole grading system:

$$S = \sum_{n=1}^{10} S_n \Big/ 10$$

eTable 1. Comparison between pregnant group and the non-pregnant group

Variables	Pregnancy (+) **(N=15021)**	Non-pregnancy (-) **(N=45627)**	P-value
Number of Times about IVF-ET	1.21±0.55	1.43±0.94	<2e-16
Age(years)	30.30±4.02	32.17±5.58	<2e-16
FSH (mIU/ml)	6.99±2.51	7.75±25.74	3e-04
AFC(n)	13.85±5.32	12.51±6.39	<2e-16
AMH	5.24±2.78	4.73±2.36	<2e-16
Inhibin B	90.16±29.39	88.25±38.47	2.41e-08
BMI (kg/m^2)	21.90±2.31	21.86±1.94	0.08
Type of infertility			
Primary	8759（58.31%）	23692（51.92%）	
Secondary	6170（41.08%）	21149（46.35%）	
Unknown	93（0.62%）	786（1.72%）	
Duration of infertility(years)	4.10±2.98	4.54±3.53	<2e-16
Number of Oocytes	11.71±5.25	11.56±7.95	0.03
M2	10.41±4.72	10.09±7.21	4.08e-07
M1	0.30±0.83	0.36±0.91	7.46e-14
GV	0.12±0.50	0.19±0.76	<2e-16
The Others$_1$	0.73±1.37	0.63±1.50	3.99e-13
2PN	7.12±3.72	6.53±5.36	<2e-16
1PN	0.46±0.81	0.42±0.81	1.51e-08
Multi PN	1.01±1.51	1.10±1.79	4.14e-08
Cleavage	8.41±4.33	7.84±6.24	<2e-16
Late Cleavage	0.44±0.96	0.52±1.18	4.44e-16
Blastulation	2.56±3.03	2.59±3.75	0.44
treatment strategy			
1- Long strategy	8887（59.16%）	26271（57.58%）	
2- Short strategy	170（1.13%）	751（1.65%）	
3- Antagonist strategy	2206（14.75%）	7365（16.14%）	
4- Ultra-long strategy	3714（24.73%）	5170（11.33%）	
5- the Others$_2$	34（0.23%）	1727（3.78%）	
6- Letrozole Microstimulation for	0（0）	3283（7.20%）	

© 2020 Liao S et al. *JAMA Network Open.*

Ovulation			
7- Ovulation induction in Luteal Phase	9（0.06%）	1060（2.32%）	
Number of Large Follicles	10.03±4.18	9.39±5.67	<2e-16
E_2	3365.59±2243.75	3670.40±2941.77	<2e-16
Progesterone(ng/ml)	1.04±0.53	1.38±32.94	0.20
Endometrial Thickness	11.60±2.31	10.80±3.05	<2e-16

Data are mean ± SD or number (percentage). BMI: body mass index (kg/m^2); FSH: follicle-stimulating hormone; PCOS: polycystic ovary syndrome; AFC: antral follicle count. "The $Others_1$" refers to the oocytes that are not at the M2, M1, and GV stages. "The $Others_2$" refers to the ovulation induction with CC (clomiphene citrate). E2, Progesterone, and Endometrial Thickness are recorded on the day of HCG administration.

eTable 2. Three grading schemes for final score of patients

Schemes	Categories	Intervals	Total sample size	Pregnancy sample size	Pregnancy rates
4 groups	D	≤2.38	3997	36	0.90%
	C	2.38~2.97	6819	693	10.16%
	B	2.97~3.66	36432	9266	25.43%
	A	>3.66	13399	5025	37.50%
5 groups	E	≤2.38	3997	36	0.90%
	D	2.38~2.97	6819	693	10.16%
	C	2.97~3.66	36432	9266	25.43%
	B	3.66~3.84	12143	4349	35.81%
	A	>3.84	1256	676	53.82%
6 groups	F	≤2.05	1686	3	0.18%
	E	2.05~2.38	2311	33	1.43%
	D	2.38~2.97	6819	693	10.16%
	C	2.97~3.66	36432	9266	25.43%
	B	3.66~3.84	12143	4349	35.81%
	A	>3.84	1256	676	53.82%

eFigure 1

© 2020 Liao S et al. *JAMA Network Open.*

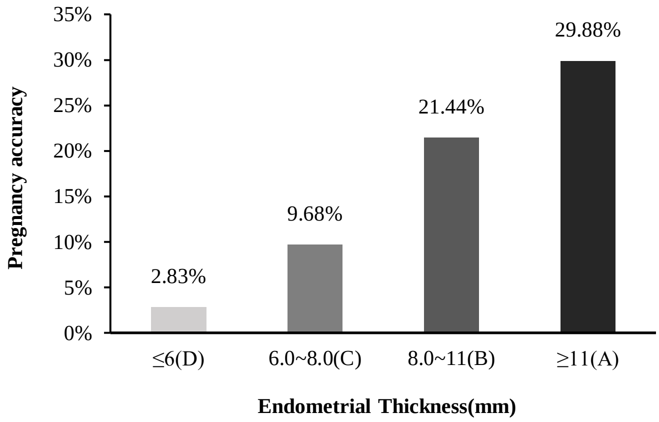

eFigure 2 The relationship between the pregnancy rate and the seven indicators

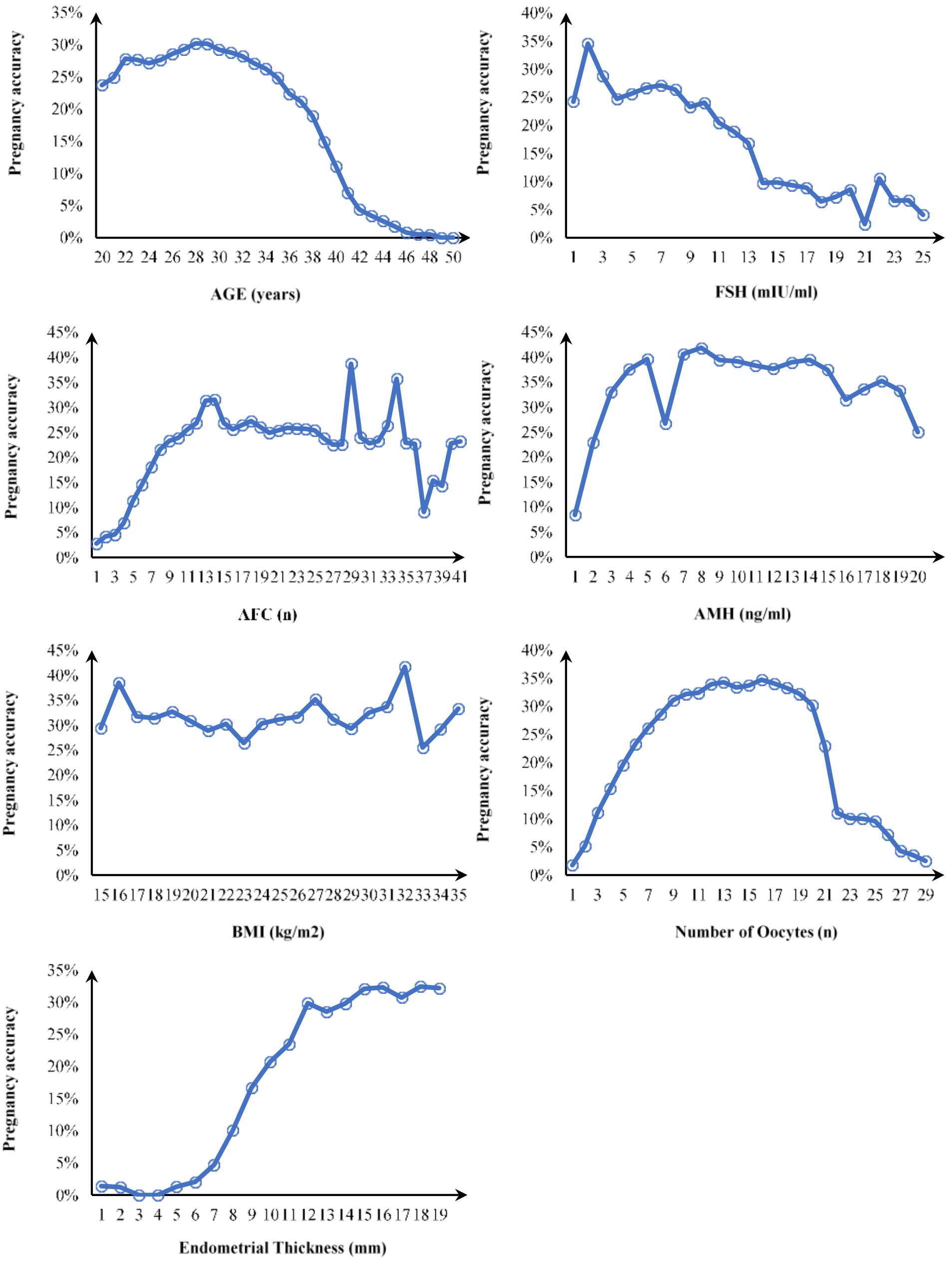

© 2020 Liao S et al. *JAMA Network Open.*
